# Alkaloids as mediators in plant-microbe interactions: Metabolism and role in the rhizosphere

**DOI:** 10.5511/plantbiotechnology.25.0310a

**Published:** 2025-09-25

**Authors:** Tomohisa Shimasaki, Ryohei Thomas Nakano

**Affiliations:** 1 Faculty of Science, Hokkaido University, Sapporo, Hokkaido 060-0810, Japan

**Keywords:** alkaloids, plant-microbe interaction, plant specialized metabolites, root microbiota

## Abstract

Alkaloids represent one of the largest classes of plant specialized metabolites, characterized by diverse chemical structures and activities. Known for their bioactive properties, these metabolites have primarily been described in the context of aboveground defense against pathogens, insects, and herbivores. Beyond these defensive functions, recent studies have revealed that alkaloids also mediate interactions between plants and their associated root microbiota. These interkingdom metabolic interactions improves plant fitness, particularly under changing environmental conditions. This review highlights the metabolism and roles of alkaloids in the rhizosphere, a critical hotspot for interactions between plants and soil microbes. We also explore key questions that expand our understanding of the role of plant specialized metabolites, extending beyond alkaloids, in plant-microbiota interactions and their broader implications for plant fitness.

## Introduction

Plants produce a wide array of structurally diverse, lineage-specific low-molecular-weight compounds known as plant specialized metabolites. While these metabolites do not directly influence plant development or reproduction, they play a vital role in host adaptation by alleviating abiotic stresses and facilitating biological interactions with surrounding organisms. Among these, alkaloids constitute one of the largest classes of plant specialized metabolites, which are defined as the group of metabolites containing at least one nitrogen atom that generally exhibit alkalinity. To date, alkaloids are estimated to comprise over 20,000 different types identified across various plant species (Yang and Stöckigt 2010; Ziegler and Facchini 2008). This extensive class of metabolites is broadly categorized into “true alkaloids” and “pseudoalkaloids” based on the origin of their nitrogen. True alkaloids, such as nicotine, camalexin, and benzoxazinoids (BXs), contain nitrogen within a heterocyclic structure derived from amino acids. In contrast, pseudoalkaloids are synthesized from non-amino acid precursors and include terpene-like, steroid-like, and purine-like alkaloids, such as aconitine, tomatine, and caffeine, respectively.

Many alkaloids exhibit potent bioactivities, including insecticidal and antimicrobial properties, and have historically been recognized as defense compounds against predators in aboveground environments (Ali et al. 2019; Khare et al. 2017; Nakayasu et al. 2018). For example, nicotine is among the most well-characterized toxic alkaloids produced by plants in the genus *Nicotiana*, which deters a wide range of insect herbivores by targeting acetylcholine receptors in the animal nervous systems (Steppuhn et al. 2004). This strong insecticidal activity inspired the development of globally used synthetic insecticides, neonicotinoids, which mimic nicotine’s mechanism of action (Elbert et al. 2008). However, recent studies have uncovered additional functional roles of alkaloids in interactions with soil-borne microbes.

In nature, plant roots interact with a diverse array of microorganisms, ranging from mutualistic to commensal and pathogenic microbes. These microorganisms form a structured community known as the root microbiota, which significantly influences plant growth and health (Durán et al. 2018). Previous research has demonstrated the critical roles of plant specialized metabolites in the interactions with the root microbiota. For instance, flavonoids and strigolactones act as signaling molecules in symbiosis with mutualistic rhizobia and arbuscular mycorrhizal fungi, respectively (Akiyama et al. 2005; Kosslak et al. 1987). In addition, metabolites, such as coumarins, flavonoids, and triterpenes, influence the colonization patterns of commensals, fostering the formation of beneficial root microbiota (Harbort et al. 2020; Huang et al. 2019; Stringlis et al. 2018; Yu P et al. 2021; Zhong et al. 2022). Recent studies have begun to elucidate the molecular mechanisms underlying plant-microbe interactions mediated by plant specialized metabolites, highlighting the crucial roles of microbial metabolism in these interaction processes (Aoki et al. 2024; Nakayasu et al. 2023; Shimasaki et al. 2021; Thoenen et al. 2024). In this review, we summarize our current understanding of alkaloid metabolism in the rhizosphere and explore the ecological implications of these metabolic interactions. Furthermore, we emphasize the necessity of investigating multiple metabolites to achieve a comprehensive understanding of the metabolic network that enacts the plant holobiont and discuss their potential applications in sustainable agriculture practices.

## Plants secrete alkaloids into the rhizosphere

Plants secrete a variety of metabolites into the rhizosphere, including both primary metabolites, such as sugars, amino acids, and organic acids, and specialized metabolites. These compounds play critical roles in initiating interactions with soil microbiota by attracting or repelling specific microbial populations and inducing gene expression related to symbiosis or pathogenicity. The levels and composition of these secreted metabolites vary across plant growth stages and are influenced by both biotic and abiotic conditions (Badri and Vivanco 2009; Stringlis et al. 2018; Sugiyama et al. 2016). Recent metabolomics studies, employing both targeted and untargeted approaches, have shown that plants secret measurable amounts of alkaloids as root exudates into the rhizosphere. For instance, Nakayasu et al., investigated the secretion dynamics of tomatine, a glycoalkaloid produced by tomato (*Solanum lycopersicum*), using a hydroponic culture system (Nakayasu et al. 2021). They revealed that tomatoes secret both tomatine and its aglycone, tomatidine, with their secretion levels varying across different growth stages.

The nutritional conditions of the soil also influence the levels of alkaloids in root exudates. For example, it has been reported that *Brachypodium distachyon* grown under low nitrogen conditions secretes reduced amounts of alkaloids and amino acids, potentially as a strategy to conserve internal nitrogen levels (Novak et al. 2024). However, the fact that the secretion of alkaloids is crucial for the defense against root pathogens and the interactions with root microbiota raises another intriguing question of how plants coordinate nitrogen storage with alkaloid-mediated root-microbe interactions, depending on the soil nitrogen conditions. In aboveground tissues, it has been shown that nitrogen status alters both plant defense response (Campbell and Vallano 2018; Rzemieniewski et al. 2024) and alkaloid metabolism (Nowacki et al. 1976; Qi et al. 2024), whereas their association remains largely unknown. Further study elucidating how plants coordinate alkaloid metabolism with root microbiota interactions in response to surrounding biotic and abiotic conditions will provide key insights into the roles of rhizosphere alkaloids in plant adaptation.

## Root microbiota affects host alkaloid metabolism

Consistent with their defensive functions, alkaloid biosynthesis is often modulated by biotic stresses such as insect and pathogen attacks (Ahmed and Kovinich 2021; Erb et al. 2012; Katoh et al. 2005). Camalexin is an indole alkaloid produced by *Arabidopsis thaliana* and related species (Glazebrook and Ausubel 1994; Khare et al. 2017). Its biosynthesis is upregulated during the activation of pattern-triggered immunity (PTI) (Nakano and Shimasaki 2024; Nguyen et al. 2022), which constitutes the first layer of the plant immune system, triggered by the recognition of microbe-associated molecular patterns (MAMPs) by cell surface-localized pattern recognition receptors. Although camalexin was initially characterized as an aboveground defense compound, Aryal et al. demonstrated that the ATP binding cassette (ABC) transporter, ABCG36, expressed in roots, exports camalexin across the plasma membrane. A mutant lacking this transporter exhibits increased susceptibility to the root pathogen *Fusarium oxysporum* (Aryal et al. 2023). Intriguingly, ABC36 also transports indole-3-butyric acid (IBA), an auxin precursor, but its transport activity for camalexin is selectively activated upon pathogen attack. This substrate specificity is regulated by QIAN SHOU KINASE1 (QSK1), a leucine-rich repeat receptor kinase. These findings in *Arabidopsis* illustrate a sophisticated regulatory mechanism governing alkaloid biosynthesis and secretion.

In addition to pathogens, nonpathogenic bacteria, including mutualists and commensals, also influence the accumulation and exudation of alkaloids. For example, rhizobia infection in the genus *Crotalaria* (family Fabaceae) roots induces nodule-specific biosynthesis of monocrotaline, a pyrrolizidine alkaloid (Irmer et al. 2015). Similarly, the exudation of camalexin from *Arabidopsis* roots is upregulated upon colonization of beneficial bacteria *Pseudomonas* sp. CH267 (Koprivova et al. 2023). Nicolle et al. uncovered the molecular mechanism by which *Streptomyces* strain AgN23, a beneficial bacterium isolated from grape roots, modulates camalexin metabolism in *Arabidopsis* roots (Nicolle et al. 2024). This bacterium produces galbonolides, which inhibit host inositol phosphorylceramide synthase (IPCS), a key enzyme in plant sphingolipid metabolism. IPCS inhibition triggers plant defense responses, including SA signaling, nuclear Ca2^+^ influx, and defense gene expression, eventually enhancing camalexin biosynthesis. Importantly, camalexin production is essential for the successful colonization of the rhizosphere by *Streptomyces* strain AgN23, illustrating novel functional aspects of camalexin as an attractant besides its role as a phytoalexin (Nicolle et al. 2024).

Systemic metabolic responses to microbiota colonization further highlight the complexity of these interactions. Korenblum et al. found that *Bacillus* colonization induces the exudation of azelaic acid (AzA) from tomato roots, which in turn systemically triggers tomatine secretion (Korenblum et al. 2020). Likewise, fusaric acid, an alkaloid produced by the fungal pathogen *Fusarium oxysporum*, induces systemic changes in tomato root exudation, including increased tomatine secretion (Jin et al. 2024). Notably, these metabolic shifts facilitate the formation of beneficial root microbiota that suppress Fusarium wilt disease. Together, these studies demonstrated that plants perceive colonization of soil-born microbes through MAMPs or microbial metabolites, which, in turn, induces shifts in host alkaloid metabolism. These shifts not only affect root microbiota colonization patterns but also drive broader ecological consequences.

## Microbial alkaloid metabolism in the rhizosphere

In addition to influencing host alkaloid metabolism, the root microbiota actively metabolizes and produces alkaloids in the rhizosphere. For instance, BXs, a class of indole alkaloids produced by *Poaceae* plants such as maize (*Zea mays*) and wheat (*Triticum aestivum*), function as defense chemicals against aboveground herbivores (Ahmad et al. 2011; Robert and Mateo 2022). In the maize rhizosphere, 2,4-dihydroxy-7-methoxy-1,4-benzoxazin-3-one (DIMBOA), a predominant root-exuded BX of maize, undergoes rapid degradation by soil microbes. DIMBOA is initially converted into methoxybenzoxazolin-2(3H)-one (MBOA) and subsequently into 2-amino-7-methoxyphenoxazin-3-one (AMPO). This catabolic activity is mediated by *Bxd* genes, which present in a subset of maize root microbiota members including *Sphingobium*, *Microbacterium*, and *Pseudoarthrobacter* (Thoenen et al. 2024). Interestingly, AMPO exhibits strong allelopathic activity, inhibiting both neighboring plant growth and bacterial growth (Thoenen et al. 2023; Venturelli et al. 2015). This indicates that alkaloid metabolism by root microbiota modulates the composition and function of alkaloids in the rhizosphere.

**Figure 1:**
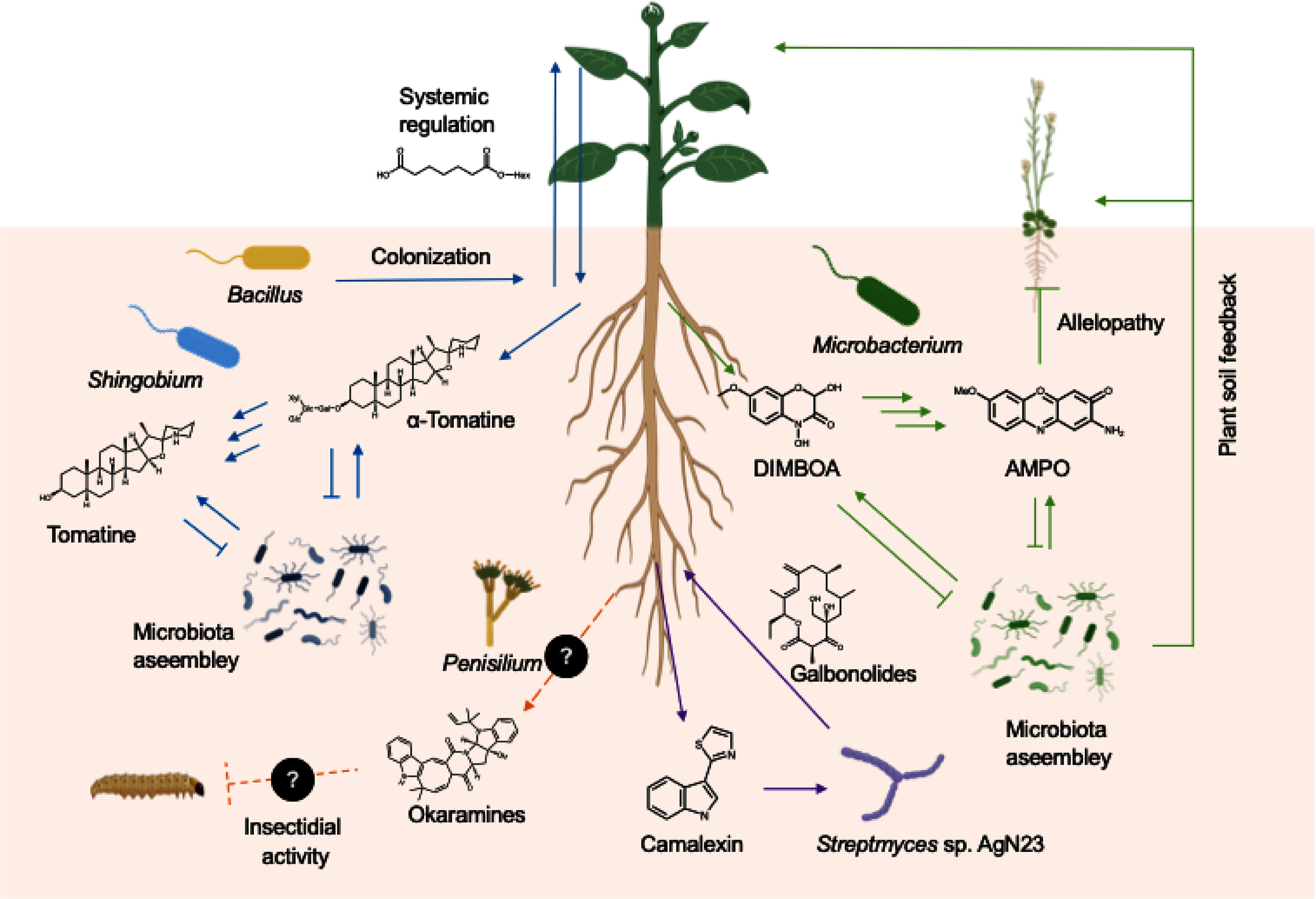
Figure 1. Schematic overview of alkaloid metabolism and role in the rhizosphere. Representatives of alkaloid-mediated plant-microbe interactions in the rhizosphere are shown. The secretion levels of alkaloids are influenced by the colonization patterns of root microbiota, and secreted alkaloids subsequently undergo microbial catabolism. Root microbiota also produce alkaloids specifically when they colonize to roots. These complex metabolic interactions underpin the structure and function of root microbiota.

An untargeted metabolomics study of the hairy vetch (*Vicia villosa* Roth ssp. villosa) rhizosphere discovered the accumulation of okaramines (Sakurai et al. 2020), a group of indole alkaloids previously identified only in soybean pulp (Okara) cultivated with *Penicillium simplicissimum* (Hayashi et al. 1989, 1991). Notably, okaramines accumulate in the rhizosphere at concentrations sufficient to exhibit insecticidal activity, suggesting that they provide resistance to insect herbivores in the rhizosphere. Also, soybean plants accumulate okaramines in their rhizosphere only when hairy vetch has been grown as a preceding crop. While it remains unclear whether okaramines accumulation results from *P. simplicissimum* colonizing soybean roots, the finding suggests that the biosynthetic potential for bioactive alkaloids may be inherited across plant generations and species through microbial associations. Similarly, *Pseudomonas koreensis* produces antibacterial tetrahydropyridine alkaloids, known as koreenceines, when they are grown in soybean root exudates (Lozano et al. 2019). This phenomenon may arise from root-secreted compounds activating alkaloid biosynthesis and/or serving as precursors for their production. Collectively, alkaloid profiles in the rhizosphere are shaped by complex metabolic interactions between plants and their root-associated microbiota (Figure 1). Characterizing the bacterial genes involved in microbial alkaloid catabolism and biosynthesis will provide keys to unraveling the complex dynamics underlying alkaloid-mediated interactions between plant and their root microbiota.

## The functions of alkaloids in the assembly of root microbiota

The secretion of alkaloids often induces shifts in the composition of root microbiota communities. Recent studies have attempted to elucidate the molecular mechanisms underlying these modulatory effects. Thoenen et al. evaluated the antibacterial activity of BXs against bacterial strains isolated from maize and *Arabidopsis* roots, finding that BXs inhibited the growth of a broad range of bacterial isolates (Thoenen et al. 2023). This aligns with the observations that the number of amplicon sequence variants whose relative abundance decreased was greater than those whose relative abundance increased in benzoxazolinone-treated soil (Schütz et al. 2021). Importantly, this study also demonstrated that maize root microbiota showed greater tolerance to BXs compared to bacterial isolates from *Arabidopsis* roots. This suggests that BXs shape the maize root microbiota by recruiting tolerant microbes and repleting intolerant soil microbiota.

Conversely, alkaloids also modulate root microbiota communities by attracting specific bacterial taxa, distinct from their defensive roles. The application of pure alkaloid compounds, such as tomatine and nicotine, modulates the soil microbial community by enriching specific bacterial taxa, including the family Sphingomonadaceae and the genus *Arthrobacter*, respectively (Nakayasu et al. 2021; Shimasaki et al. 2021). Notably, *Sphingobium* and *Arthrobacter* are predominant in the rhizosphere of tomato and tobacco plants, and bacterial strains isolated from these roots exhibited the ability to degrade tomatine and nicotine, respectively (Nakayasu et al. 2023). Furthermore, the reduced relative abundance of Sphingomonadaceae in a low tomatine-producing tomato mutant compared to a wild-type plant underscores that root microbiota utilizes host-produced alkaloids as nutrient sources to adapt to the rhizosphere environment. It is noteworthy that a single alkaloid plays a dual role, either attracting or repelling the soil microbiota members, with its effects varying depending on the bacterial strain. In fact, both nicotine and tomatine are also known to exhibit antibacterial activity (Kaup et al. 2005; Pavia et al. 2000; You and van Kan 2021). BXs serve as nutrient sources and chemoattractants for maize root microbiota (Neal et al. 2012; Thoenen et al. 2023) besides their antimicrobial properties. Together, these studies illustrate the role of alkaloids in the assembly processes of root microbiota, facilitating the selection of soil microbiotas that are metabolically adapted to these compounds.

## Alkaloids trigger the beneficial function of root microbiota

In addition to their direct role in improving plant survival by deterring natural enemies, recent studies reveal that alkaloids indirectly contribute to plant fitness by inducing beneficial activities of the root microbiota. A genome-wide association analysis using 172 *Arabidopsis* accessions identified an association between sulfatase activity in the rhizosphere and the *CYP71A27* gene, which encodes an uncharacterized cytochrome P450 enzyme (Koprivova et al. 2019). This gene appears to be involved in camalexin biosynthesis in roots. Knock-out mutant of *CYP71A27* reduced sulfatase activity in the rhizosphere and lost the plant growth-promoting effects provided by *Pseudomonas* sp. CH267. Importantly, camalexin did not affect the growth or colonization pattern of CH267, suggesting that its influence is limited to modulating the functional behavior of the strain CH267 rather than accommodating its presence.

Hu et al. found that maize grown in soil previously cultivated with maize increased insect resistance, though its growth was inhibited (Hu et al. 2018). This activity, often referred to as a “soil legacy effect” or “plant-soil feedback”, reflects the ability of plants to influence the performance of the next generations growing in the same soil by altering its abiotic and biotic properties (Bakker et al. 2018; Janse van Rensburg et al. 2024; Rolfe et al. 2019). Notably, this feedback effect was suppressed when the legacy soil was conditioned by BX-deficient mutant plants or sterilized before replanting the next generation of maize. However, the effect was restored when soil conditioned by BX-deficient mutants was chemically complemented with 6-methoxy-benzoxazolin-2-one (MBOA), a breakdown product of BXs. While it remains to be carefully assessed whether BX-mediated feedback is achieved through the recruitment of specific beneficial bacteria or by altering the entire root microbiota community, these studies demonstrate that BXs induce plant-soil feedback via the root microbiota.

Collectively, these studies illustrate that alkaloids trigger the beneficial functions in the root microbiota by 1) inducing the beneficial activity of specific bacteria and/or 2) modulating the colonization patterns of the root microbiotas. However, despite evidence that bacterial adaptation to host alkaloids is crucial for root microbiota members to preferentially colonize their host, the functional significance of these metabolic traits from the host plant’s perspectives remains poorly understood. This raises the intriguing question of whether microbiota adapted to metabolize host alkaloids inherently benefit the host plant. Recent technical advances, including the establishment of root-derived bacterial culture collections, allow us to reconstruct root microbiota through inoculating multiple bacteria cultures into gnotobiotic plant culture systems (Chesneau et al. 2025; Durán et al. 2018; Liu et al. 2019). Using the synthetic community (SynCom) approach, we can now design desired bacterial communities and evaluate their impacts on various plant hosts under controlled conditions (Wippel et al. 2021). Using this methodological approach, Zhou et al. demonstrated the functional connection between the metabolic traits of root microbiota and their beneficial effects (Zhou et al. 2024). They designed two SynComs consisting of bacterial strains metabolically adapted or non-adapted to Amaryllidaceae alkaloids (AAs), a group of specialized metabolites unique to Amaryllidaceae plants such as *Lycoris radiata*. Their findings revealed that inoculation with the adapted SynCom, but not the non-adapted SynCom, increased the accumulation of AAs, which, subsequently, enhanced plant resistance against a fungal pathogen. Bridging the gap between the metabolic and functional traits of root microbiota is necessary to understand how plants adapt to their environments by establishing metabolic networks with their root microbiota.

## Conclusion and future perspectives

Over the past decades, many studies have demonstrated the crucial role of plant specialized metabolites in plant-microbe interactions. However, most of these studies have focused on a single class of metabolites, despite plants secreting over a thousand metabolites into the rhizosphere (McLaughlin et al. 2023; van Dam and Bouwmeester 2016). It therefore remains unclear how plants coordinate the functions of root microbiota through multiple specialized metabolites that differentially affect root microbiota communities. In this last section, we emphasize the need for a comprehensive framework to decipher the metabolic networks underlying the structure and function of plant root microbiota, extending beyond alkaloids.

**Figure 2:**
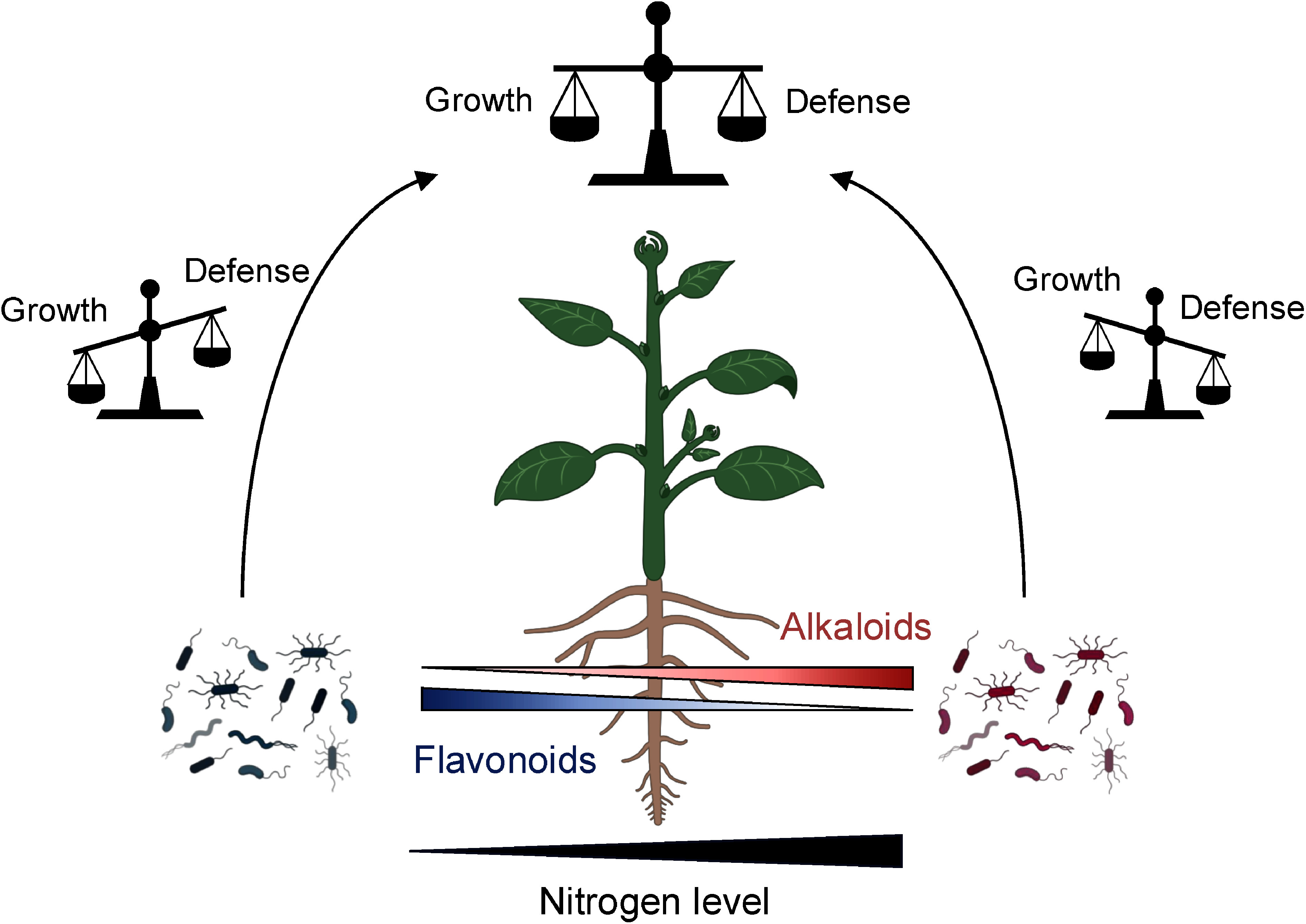
Figure 2. Proposed model of the crosstalk between alkaloid and flavonoid metabolic pathways coordinating growth-defense trade-offs via root microbiota. Plants adjust the secretion levels of flavonoids and alkaloids depending on the soil nitrogen availability. Flavonoids are actively secreted under the nitrogen deficiency to form the beneficial microbiota which improve the nitrogen acquisition. In contrast, plants restrict alkaloid secretion to conserve internal nitrogen levels, although they enhance herbivore resistance by triggering the microbiota function. These observations imply plants coordinate the function of root microbiota to balance the growth and defense through flavonoid and alkaloid metabolism.

Plant specialized metabolites are classified into three major groups, alkaloids, phenolics, and terpenoids, each synthesized via different metabolic pathways (Huang and Dudareva 2023). In order to deepen our understanding of how these metabolic pathways interact and their ecological consequences in the interaction with root microbiota, we propose that exploring the trade-off between alkaloid and flavonoid metabolism offers a promising model. Flavonoids, widespread polyphenolic compounds in the plant kingdom, often mediate interactions with microbes that alleviate nitrogen deficiency (Sugiyama and Yazaki 2014; Wang et al. 2022). For instance, leguminous plants secrete flavonoids to initiate symbiotic nodulation with rhizobia by inducing nodulation gene expression (Firmin et al. 1986; Kosslak et al. 1987). In addition, flavones secreted from maize roots enrich the Oxalobacteraceae family, which improves nitrogen acquisition by promoting lateral root development (Yu P et al. 2021). Importantly, plants increase flavonoid accumulation and secretion under nitrogen-deficient conditions (Sugiyama et al. 2016) while reducing alkaloid production. This suggests a growth-defense trade-off between alkaloid and phenylpropanoid pathways, wherein plants allocate limited resources to either growth or defense based on the surrounding environments. Given that soil microbiota conditioned by either alkaloids or flavonoids enhance herbivore resistance and nutrient acquisition, it is plausible that plants balance growth and defense by coordinating the microbial functions through these metabolic pathways (Figure 2).

The metabolic traits of root microbiota also highlight the importance of examining multiple metabolites from a different perspective. We previously demonstrated that *Arthrobacter* strains isolated from tobacco roots could degrade unrelated classes of tobacco-specific specialized metabolites, nicotine and santhopine, whereas strains originating from other environments degraded neither or only one of these metabolites (Shimasaki et al. 2021). These findings suggest the cooperative action of multiple metabolites in bacterial adaptation to the host rhizosphere. However, significant knowledge gaps remain regarding the functions of plant specialized metabolites, both as individual components and as a cocktail of these metabolites. In a study focusing on plant primary metabolites, Zhalnina et al. demonstrated an association between the metabolic capacities of host metabolite mixtures and the colonization preferences of root microbiota (Zhalnina et al. 2018). Integrating exometabolomics and comparative genome analysis approaches, they showed that bacterial isolates enriched in the rhizosphere of *Avena barbata* preferentially consumed multiple primary metabolites from root exudates compared to non-adapted isolates. Recently, similar culture-dependent approaches utilizing plant tissue extracts or root exudates have also been employed to study bacterial growth and transcriptomic responses to plant specialized metabolites (Unger et al. 2024; Yu K et al. 2021). Compared to canonical approaches using a single metabolite as a sole carbon source, metabolite cocktails better mimic the chemical environments of the rhizosphere while retaining its complexity. Combining these culturing methods with omics techniques, including transcriptome and exometabolomic analysis, not only enables us to analyze bacterial responses to multiple different metabolites but also helps identify novel compounds driving these interactions.

Lastly, the combinatorial effects of multiple specialized metabolites may offer strategies for leveraging root microbiota functions in sustainable agriculture. In field conditions, the inoculation of beneficial microorganisms often exhibits instability and low persistence, likely due to competition with indigenous microbes. Engineering beneficial bacteria with gene clusters that catabolize host-specific metabolites could enhance colonization stability by conferring a competitive advantage (Haskett et al. 2021). Utilizing multiple specialized metabolites in such strategies may improve the selectivity of these interactions, allowing us to design the function of root microbiota. Root microbiota is functionally inseparable from host plants, forming an ecological unit often referred to as the “holobiont” (Hassani et al. 2018; Theis et al. 2016). Plant specialized metabolites play pivotal roles in organizing and functionalizing plant holobionts. Adapting a holistic perspective to elucidate the complex metabolic interactions within plant holobionts will be crucial for harnessing their potential in sustainable crop production.
